# Using occupational therapists in vocational clinics in primary care: a feasibility study

**DOI:** 10.1186/s12875-020-01340-5

**Published:** 2020-12-13

**Authors:** Avril Drummond, Carolyn Coole, Fiona Nouri, Jo Ablewhite, Genevieve Smyth

**Affiliations:** 1 School of Health Sciences, University of Nottingham, Medical School, Queens Medical Centre, Nottingham, NG7 2UH UK; 2 Royal College of Occupational Therapists, 106-114 Borough High Street, London, SE1 1LB UK

## Abstract

**Background:**

GPs are under considerable pressure providing routine care. However, they may not be the most appropriate professionals to manage getting patients back to work, and keeping them there.

**Objective:**

To test the feasibility of delivering occupational therapy-led vocational clinics (OTVoc) to provide return to work advice and support for people with musculoskeletal conditions and mental health problems, in primary care.

**Methods:**

Prospective mixed methods study in two primary care centres (eight GP surgeries). We collected anonymised service level data on all patients receiving OTVoc. Next, patient participants who met inclusion criteria and consented, undertook baseline and 3-month follow-up assessments. Interviews were also conducted to explore stakeholders’ views- GPs, Nurse Practitioners, Front Desk Staff, Occupational Therapists, patients and their employers about OTVoc- and included study eligibility, referral, experiences and attitudes to return to work. Data were analysed using descriptive statistics and thematic analysis.

**Results:**

The majority of standardized measures showed some improvement over the study period: the sickness absence rate dropped from 71 to 15% and use of GP ‘fit’ notes reduced from 76 to 6%. Interview data indicated positive attitudes to OTVoc, the use of the fit note and the Allied Health Professions Health and Work Reports (AHP H&WRs). GPs felt that OTVoc reduced their workload.

**Conclusion:**

Further research is feasible and warranted. OTVoc was positively received and stakeholders believed it was effective in getting patients back to work or preparing for their return. There was enthusiasm for extending service eligibility criteria, suggesting potential for further development and evaluation.

## Key messages

- GPs may not be the most appropriate professionals to manage back to work issues
- OTs already have the therapeutic skills to deliver this advice and support.
- Patients felt the OTs had more time to deal with their work issues
- Sickness absence rate reduced over the study period
- GPs, other staff and employers regarded the OT input positively

## Lay summary

We wanted to test whether an Occupational Therapy-led vocational clinic (OTVoc), to provide return to work advice and support for people with work problems, in primary care, was feasible.

We conducted a study at eight GP surgeries. We examined anonymous data and conducted a mixed methods study. Patient participants, who consented, undertook baseline and 3-month follow-up assessments. Interviews were also conducted to explore the views of stakeholders about the clinics. These included GPs, Nurses, Front-desk Staff, Occupational Therapists, patients and their employers.

The majority of measures showed some improvement: the sickness absence rate dropped from 71 to 15% and use of GP ‘fit’ notes reduced from 76 to 6%. Interview data indicated positive attitudes to the clinics and GPs felt that OTVoc reduced their workload.

OTVoc was well received and stakeholders believed it was effective in getting patients back to work or preparing for their return. There was a suggestion that OTs were better placed and had more time than GPs to deal with health-related work issues. There was enthusiasm for extending service eligibility criteria. The results suggest there is potential to change current management of vocational issues in primary care and for further development, research and evaluation.

## Background

Work is important for good physical health, mental health and well-being [1]. It is therefore essential that patients are supported to undertake work, even if they cannot perform at full capacity. However, it is widely acknowledged that many do not receive the help they need to stay at work or to make a successful, sustained return [2, 3].

Evidence shows that healthcare for musculoskeletal (MSK) conditions and mental health conditions should be work-focused [4]. Early intervention is essential to prevent long-term sickness absence, and should utilise a ‘stepped-care approach’ ranging from low-intensity interventions which will be adequate for most sick or injured workers to more intensive interventions for those needing more help to return to work. Good communication and coordination between the individual worker, healthcare practitioners, and the workplace are required.

In the UK, there are currently two systems through which advice regarding a person’s fitness for work may be communicated to their employer. The Statement of Fitness for Work (known more commonly as the ‘fit note’) replaced the ‘sick note’ in 2010 [5] and is completed by doctors to provide advice about fitness to work. The Allied Health Professions Health and Work Report (AHP H&WR), which was also introduced in 2010, is similar to the fit note, but is completed by Allied Health Professionals (AHPs). Both systems can be used to claim sick pay from an employer, however only the fit note can be used to claim UK state benefits.

Sickness certification is a huge task for doctors, and particularly those in primary care; in 2017, over a 3-month period, over 1.3 million certificates were issued by 61% of UK GP practices in England [6]. Yet previous research found that many GPs were not clear how to complete the Statement of Fitness for Work (‘fit note’) and did not feel they had enough training around this [7]. This may be why only 7% of Fit Notes have the ‘may be fit’ option selected, and why advice provided is limited [6, 7]. It may also be because some GPs do not regard issuing fit notes as part of their role [8]. However, people continue to turn to their GP regarding work issues.

AHPs are well placed to provide support around work and workplace modifications. They can use the AHP H&WR [9] to provide detailed information for the individual, employer and GP on the effects and impact of work-related difficulties. This can include specific recommendations such as changes to working hours, workplace modifications, and the need for specialist equipment. In 2017 [10], the UK government signalled its intention to reform the existing fit note and to prepare for legislation to extend sickness certification to other healthcare professionals.

The purpose of the study was to find out how an occupational therapy-led return to work advice and support clinic (OTVoc), in primary care, might help patients to return to, and remain in, work. The main objective was to test the feasibility of delivering OTVoc to people with MSK conditions and mental health problems: these conditions were specified by the funding body as they are the main reasons for sickness absence (after coughs and colds) [10]- in primary care. However, in addition, we wanted to collect specific data from a nested research component in order to determine if a future definitive trial would be feasible. We therefore also collected data on: recruitment/retention; the feasibility of collecting self-reported data; patient and stakeholder views about OTVoc.

## Method

This was a mixed- methods study with a sequential design based in two-centres, Solent GP surgeries and South Pembrokeshire GP Cluster, covering eight surgeries in total, conducted from January (recruitment from March) to November, 2019. Four OTs, who were funded by the research grant, were employed specifically to provide support to patients with work-related problems. The four OTs were employed because of their knowledge of getting people back to work. However, in addition, they were provided with core initial training from a Consultant OT specialising in return to work issues and they were encouraged to network and exchange tips and good practice with each other throughout the study. Patients discussed their situation with the OTs and most were provided with personalised self-management techniques and advice about work adjustments/modifications. Patients were offered the option of direct liaison between the OT and their employer to implement these.

There were three parts to the study. We collected 1. data about all patients attending the OTVoc service and, from those who then provided informed consent, we conducted research involving 2. a questionnaire evaluation of OTVoc and 3. interviews with patients, staff and employers. Thus:

1. We collected anonymous **service level data** on patient demographics, referral details, intervention details and usage of fit notes and/or AHP H&WRs in order to understand the characteristics of patients attending the clinical service. In order to be eligible for OTVoc, patients had to be registered with the practice; aged between 18 and 80; in employment, including self-employment; self-identifying as having a mental health and/or MSK condition; self-identifying as struggling with employment; currently at work/on sickness absence. Patients were not able to participate if they required urgent medical attention; were unemployed; required evidence for welfare benefits (such as for Personal Independence Payments), or for litigation against their employer in work-place tribunals.

2. **Questionnaire evaluation**- We collected data from people attending the service, who consented, in order to measure recruitment and retention rates to this aspect of the research and investigate the feasibility of collecting patient self-reported data to inform a future trial. As this was a feasibility study, formal sample size calculations were not required. A target number was based on time/resources and on similar research [11] and it was estimated it would be possible to recruit 50 patients to the research.

Participants were identified when referred/self-referred to OTVoc and had to be able to give informed consent. Patients were not able to participate if they were unable to read/speak English/Welsh or unable to complete paper-based questionnaires.

If a patient was eligible, the OT sent an information sheet and took verbal consent. At the first appointment, written consent was secured and baseline questionnaires were completed. A postal follow-up questionnaire and SAE were sent 3 months later with a further reminder after 4 weeks.

The following demographic/baseline data were collected: year-of-birth; gender; self-identified health problems affecting work; current work situation; usual number of hours/worked; number of sick days in previous 3 months. Follow-up data were collected 3 months later: self-identified health problems affecting work; details of changes in work situation; current work situation; usual number of hours/days worked; number of sick days in previous 3 months; number of AHP H&WRs completed.

Standardized measures collected at baseline and follow-up included:

- Perceived expectations of Working Usual Hours and Duties in 3 months’ [12]
- Perceived current ability to work- Work Ability Scale [13]
- Belief in ability to deal with situations generally using the General Self-Efficacy Scale [14]
- Perceived mental wellbeing using the Warwick-Edinburgh Mental Well-Being Scale [15]
- Perceived quality-of-life- Euroquol 5D-5L [16, 17]
- Perceived Readiness for Return to Work Scale [18] - measures *not back at work* under four headings: ‘Precontemplation’, ‘Contemplation’, ‘Prepared for Action – self-evaluative’ and ‘behavioural’ and *back at work:* ‘Uncertain Maintenance’ and ‘Proactive Maintenance’.
- Return to Work Self-Efficacy Questionnaire to measure belief in ability to deal with work situations [19]
  Group means were imputed for individual missing questions. Data were analysed descriptively.

**3. Interviews-** to explore stakeholders’ and patients’ views about OTVoc.

Staff and patients who had participated in the questionnaire evaluation study were invited to take part in telephone interviews, as were employers of patients who had given consent for this. All interviews were conducted by one researcher (FN) an OT by professional background, who followed an interview schedule and covered key areas: awareness of the OTVoc service; referral and access to the service; communication between staff, patients and employers; information on OTVoc interventions; use and value of the AHP H&WR and both positive and negative opinions of OTVoc generally. This ensured consistency both in the approach taken and in the questions asked. Views were also sought on any potential improvements or recommendations which the participants thought could be made. Verbal consent was taken. Interviews were digitally recorded, transcribed in full by an independent transcription service and then checked for accuracy by the researcher conducting the interviews (FN). Two researchers (CC and JA) read each transcript and independently developed, then agreed, a coding framework [20]. One team member (FN) then entered the transcripts into a qualitative data package (NVivo 10, QSR International) and coded each transcript. Overarching themes were identified independently by three researchers (FN, CC and JA), then discussed and agreed with the wider research team, and the data were then mapped on to the interview schedule.

## Results

### The OTVoc service

One hundred fifty-eight referrals were made to the service of which 22 did not meet service eligibility criteria. Of those not eligible, 19 were not in work, two did not have an MSK or mental health condition, and no reason was recorded for one.

Of the 136 referrals which were accepted, about half came from self-referral. Most were aged 31–50 and two-thirds were women. Similarly, two-thirds had mental health problems either alone or alongside a MSK condition, and worked between 26 and 38 h a week. Participants were employed in a variety of jobs and included: teacher, postman, care worker, GP, cleaner, shop-assistant, auditor, HGV-driver, nurse, bartender and poultry-worker.

At referral, two-thirds were on sickness absence and the rest were struggling at work with no workplace modifications. About three-quarters had used a “not fit” GP fit note in the previous 3 months. Almost a fifth (17%) had been issued with four or more “not fit” GP fit notes in the past 3 months. Table 1 shows work status on referral and discharge.

**Table 1:** Work status on referral and discharge for 136 patients attending OTVoc

	At referral		On discharge	
	n	%	n	%
Off sick	97	71.3	21	15.4
At work with adjustments	7	5.2	67	49.3
At work no adjustments	32	23.5	35	25.7
Unemployed	0	0	5	3.7
Other	0	0	1	0.7
*Not known*			*7*	*5.2*
**Total**	136		136	

81% (*n* = 110) had at least one face-to-face session. Average overall duration of clinic time per patient was 132 min and was associated with an average of 35 min ‘admin’.

Prior to referral, 104 patients (76%) had been issued with a ‘not fit’ fit note in the previous 3 months. The greater number were 1 note, but 23 (17%) were issued with 4 or more. In the subsequent 3 months after referral, 42 (31%) were issued with ‘not fit’ fit notes and 12 (9%) with ‘may be fit’ notes.

OTs issued ‘not fit’ AHP H&WRs to 24 (18%) and ‘may be fit’ AHP H&WRs to 72 (53%). By comparison, post referral, 8 (6%) were issued with both ‘not fit’ fit notes and ‘not fit’ AHP H&WRs. Seven patients (5%) were issued with both ‘may be fit’ fit notes and ‘may be fit’ AHP H&WRs.

### Questionnaire evaluation

Fifty-two patients were recruited across the 2 centres, 37 (71%) and 15 (29%), between March and July 2019 for this part of the study. All completed baseline questionnaires and 30 returned these 3 months later. Almost three-quarters were female and three-quarters had a mental health condition (either singly or in combination with a MSK condition), as in the larger group, but they were younger (29–38 years of age). Half (50%, *n* = 26) were on paid sick leave, and just over one-third (35%, *n* = 18) were at work in their usual job at baseline (see Table 2). More than half (60%) were employed 5 days/week. On average, participants had taken 17 sick days in the previous 3 months.

**Table 2:** Work Situation of participants who agreed to complete a questionnaire evaluation at recruitment (*n* = 52) and at discharge (*n* = 30) from OTVoc

WORK SITUATION	ON RECRUITMENT TO OTVoc n = 52		ON DISCHARGE FROM OTVoc n = 30	
	n	%	n	%
**In usual job**	**18**	**34.7**	**14**	**46.7**
**Reduced hours**	**0**	**0**	**1**	**3.3**
**Reduced duties**	**0**	**0**	**1**	**3.3**
**Reduced hours and duties**	**0**	**0**	**1**	**3.3**
**Paid sick leave**	**26**	**50.0**	**5**	**16.8**
**Unpaid sick leave**	**6**	**11.5**	**2**	**6.7**
**Paid leave/holiday**	**1**	**1.9**	**1**	**3.3**
**Unemployed**	**0**	**0**	**1**	**3.3**
**Other**	**0**	**0**	**3**	**10.0**
***Not known***	***1***	***1.9***	***1***	***3.3***
**TOTAL**	**52**		**30**	

At baseline, 20 (38%) reported they were at work; 32 (62%) reported they had not yet returned. The participants were from a range of jobs such as: motor mechanic, carpenter, psychiatric nurse, dietician, café assistant, shop manager, nursery worker, accounts assistant, benefits manager and linguist/translator. A summary of key results from baseline may be seen in Table 3.

**Table 3:** Results for participants attending OTVoc, who completed a questionnaire evaluation at recruitment and at follow-up

		BASELINE		FOLLOW UP	
Name of scale	Scale range	Number completing	Mean (Standard deviation) Range	Number completing	Mean (Standard deviation) Range
**a. Expectations of working** usual hours and duties in 3 months’ time	0–10	52	6.62 (3.08) 0–10	29	6.76 (3.66) 0–10
**b. Perceived current work ability**	0–10	52	4.17 (2.49) 0–10	29	6.28 (3.17) 0–10
**c. General Self-Efficacy**	10–40	52	26.67 (5.87) 10–37	29	29.28 (4.93) 19–40
**d. Warwick-Edinburgh Mental Wellbeing**	14–70	52	36.17 (8.30) 18–57	29	45.24 (9.49) 27–70
**e. EQ 5D5L Index** ^b^	−0.594 – 1.00	51	0.53 (0.28) −0.35 – − 0.85	29	0.64 (0.26) − 0.05 – 1.00
Health today (Visual Analogue Scale)	0–100	51	41.96 (18.63) 4–70	29	60.07 (26.72) 5–95
**f. Readiness for work**- **(not returned)** Precontemplation	3–15	32	6.16 (2.81) 3–15	9	7.44 (3.64) 3–14
Contemplation	5–15	32	10.75 (2.33) 5–14	9	9.00 (3.81) 5–14
Prepared for Action–self evaluative	4–20	32	9.81 (3.50) 4–17	9	10.00 (3.90) 4–17
Prepared for Action –behavioural	3–15	32	10.16 (2.59) 3–15	9	10.11 (3.89) 3–15
**Readiness for work**- **(returned)** ^a^ Uncertain maintenance	5–25	20	19.25 (3.97) 10–25	20	14.50 (4.98) 7–24
Proactive maintenance	4–20	20	15.90 (2.38) 11–20	20	16.90 (2.65) 9–20
**g. Return to Work Self-Efficacy**	11–66	52	39.58 (10.59) 11–64	29	43.04 (9.91) 24–59

^a^ For this subscale, a lower score denotes better performance. For all other scales, a higher score is better

^b^ Due to admin error, anxiety and depression questions were omitted and mean scores were imputed

At follow-up, 30 questionnaires returned: 23 (77%) were from one centre and 7 (23%) from the other. One questionnaire was not completed, aside from written comments. Three-quarters (73.3%) reported that health problems were still affecting their work. On average, participants had taken 35 sick days in the previous 3 months. Almost half (46.7%) were working in their usual job. See Table 2.

Just over three-quarters (76.7%) had received at least one AHP H&WR while using the OTVoc service. Almost two-thirds (63.3%) had shared their AHP H&WRs with their workplace and just over one-third (36.7%) had used it for sick pay purposes. Follow up results for five of the self-reported scales are shown in Table 3**.** Here it can be noted that overall, at 3 months after referral, sickness absence had reduced from 50 to 17% and most scales indicated a slight improvement -particularly for perceived work ability, mental wellbeing and general health.

### Interview study

#### Participants

Fourteen patients and twelve stakeholders were interviewed. Patient interview duration ranged from 18 to 50 min (mean 27 min): other interviews ranged from 10 to 54 min (mean 24 min). The patients interviewed had a wide range of health problems affecting their work including anxiety, depression, low back pain, stress, knee/ foot problems, fatigue, bladder problems: the majority had more than one problem. The stakeholders included GPs (*n* = 2), a nurse (*n* = 1), front-desk staff and practice managers (n = 2), OTVoc OTs (*n* = 4), and patient employers (*n* = 3). The patients came from a range of jobs including community carer, teacher, librarian, HGV driver, maintenance technician, and park ranger.

#### Key themes

These were ‘Access to OTVoc’, ‘Communication’, ‘Integration within the GP practice’, the ‘AHP H&WR’ and ‘Evaluation’.

##### Access to OTVoc

Practice staff felt referral was straightforward. OTs appreciated support from practice managers, such as attaching OTVoc leaflets to fit notes to facilitate self-referral. OTs preferred self-referrals as they perceived that these patients were more motivated. However, they also received direct referrals.

*I think I sent quite a lot of patients with back pain, orthopaedic stuff, and I think I have a patient who’s having quite a lot of work-related stress, which needed just a bit more unpicking and more time than I could give it.* ST008 (GP)

Overall, patients appreciated the accessibility and availability of OTVoc. Even those who had not returned to work by the end, felt they had made progress. They found the mental health support and employer mediation important in building confidence and skills. Signposting to information support and exploring workplace modifications/ alternative options were also valued. Many appreciated not feeling time pressurised as clinics were flexible and relatively open-ended.

*Yeah, I mean the GPs I think the longest I’ve ever been with one has been 15, maybe 20 minutes at the most at the surgery. With obviously seeing [the OT] it was a relief to know I had definitely an hour.* W0069 (Patient)

##### Communication

Practice manager support was crucial for OTs in gaining access to systems and in getting GPs and staff on-board. GPs described how contact with the OTs informally through ‘corridor conversations’ made them feel confident to refer.

*Yeah, it’s kind of in the corridor and it’s corridor passing or sticking your head in to the room and updating me or me just saying oh I’ve sent this patient to you, can you look out for them, and then obviously in the notes as well.* ST009 (GP)

The OTs also found being able to talk to practice staff informally valuable for eliciting and sharing information. Communication between the OT and employer was dependent on the patient’s wishes but when direct contact was made, this was viewed very positively by employers.

*I think what I gained from it was a little bit greater insight into some of the challenges that my employee had faced. I found it actually very constructive. We had a very open conversation. She obviously trusts the OT.* EM002 (Employer)

##### Integration within the GP practice

The OTs enjoyed working at the practices. Being located within the same building made patient access easier and enabled the OTs to make staff aware of their presence. OTs described GPs as being very busy with back-to-back appointments with few, if any, breaks. OTs were perceived to have time to ask more questions, and could approach mental health concerns more easily than GPs within the constraints of their 10-min slots. Staff were happy to refer to OTVoc and were confident in the care patients received.

*… . and I think it’s reducing workload, but also improving the quality of care which is I think why I was brought into the surgery for the mental health background. It’s not only, I’m reducing the workload of GPs by taking on those patients but we’re giving better quality care, and that’s what I’m really focused on. And that’s why it was really exciting to have [the OT] there, because I can only do so much.* ST002 (practice nurse)

*And I think it would grow a lot. I mean we’re getting a lot more, trying to get more physios into primary care, which is making a difference. And I think certainly OTs would, I personally think they would make a massive impact in primary care*. ST008 (GP)

The OTs were positive about their experiences and the value of OTVoc, feeling their training in assessment and problem-solving made them ideally placed to offer vocational support and advice. OTs described receiving positive feedback and believed OTVoc had been successful.

*My experience as an occupational therapist working in this clinic has been really exciting actually, because we’ve been able to spend quality time with the people that have been referred to us. Giving a really holistic intervention and following them up, reviewing them and seeing progress along the way, helping people back into work. But it’s not just been about work, because we’re occupational therapists, so we look at the whole person. So, it’s been really exciting to see people make changes to their health and wellbeing as well which has impacted upon how they’re managing at work day to day.* ST001 (OT)

##### AHP H&WR

Employers varied in their experiences of the AHP H&WR. One reported that they would accept this as long as it was an official document issued by the GP practice. GPs reported that the information in the AHP H&WR informed their completion of a fit note and thought the OTs had a better understanding of workplaces.

*And then I they sorted out doing one of those fit notes that the OTs can do, where they can literally do, it’s like our sick notes but.****..*** *Yes, I’m sure there’s a name for it that I’ve forgotten what it is … … … and I thought it was brilliant that they could do that, that they could actually have their own little recommendations and that they could liaise with employers if patients were keen. I just thought it just added a really nice extra bit, because we don’t, as a doctor, you know, the patients come in and they say I do this job, and I sit there going well I don’t even know what that is, I don’t know what that means.* ST008 (GP)

Practice staff and OTs generally believed that OTs should have the authority to sign fit notes as this would simplify the process, save GP and patient appointment time and increase the credibility of OTs. However, a joint approach with the GP might be required in complex cases: one stakeholder questioned whether OTs had sufficient skills to assess the clinical implications of a patient’s diagnosis.

OT interviewees believed the AHP H&WR was a useful tool, although they were unsure whether all employers would accept it as evidence for sick pay purposes. Some patients had also requested a fit note as they were receiving Universal Credit whilst working, and could not use the AHP H&WR for this purpose. Although some patients reported that their employer had questioned it initially, after making enquiries they were happy to accept it for sickness absence purposes.

*Yeah, I took a copy for myself and gave them to my employer. And she said well I’ve never seen one like this before, I’d better double check with the operations manager to see if we can accept it. And when she came back, she said oh yes, we can, it’s just obviously they’re not very popular so to speak, because everybody just deals normally with the GP.* W006 (patient)

A practice coordinator suggested that the lack of AHP H&WR awareness amongst employers and practitioners needed addressing.

##### Evaluation of the service

Employers described OTVoc as positive and, in particular, noted the mental health support provided, the practical nature of the intervention, and the time OTs had available.

Practice managers were generally supportive of OTVoc and viewed it as valuable in addressing work issues that GPs were unable, or resistant, to manage. They were not convinced that it actually reduced GP workload, but saw it as ‘new work’.

However, GPs and a practice nurse felt OTVoc had definitely reduced GP workload. The GPs reiterated the idea that the OT was raising issues they could not, due to time restrictions: they saw the potential to refer on to be of real value.

*I don’t have any great gripes or worries. I think it’s been a good service and I’d like it to continue. I think she’s been brilliant. It’s a service that there isn’t really anyone else doing for patients really. And there are some that are really quite complex and she’s done brilliant work with. So, no I don’t have anything bad to say, I think it’s all good.* ST009 (GP)

Most patients valued the opportunity to access a professional with time to listen. The provision of signposting to relevant information or support were also seen as important. OTVoc was viewed as invaluable by most as it helped them re-evaluate, appreciate alternatives they may not have considered (e.g. reducing working hours) and, frequently, to gain confidence to return to work. Many considered that without OTVoc they would not have returned. It was also considered helpful to have the OT’s recommendations summarised in the AHP H&WR for the employer. Through the OT, one employer put adjustments in place that a patient reported requesting for many years.

*But I just found the whole experience very therapeutic and like I say and actually forcing me to look in other directions to, you know, just looking at the bigger picture really than, I’m in pain, this is what’s going to happen. It broadened my field of vision really.* W032 (patient)

There was little negative reporting. One employer was disappointed not to have a face-to-face meeting with the employee and OT. One OT felt they did not receive enough referrals, but believed this would improve with increased OTVoc promotion. Another OT cited the lack of space in the surgeries as restrictive. One of the GPs lamented that the service was only a pilot.

*No, I think it’s good. I would like it to be an ongoing thing though, the pilot thing, because I think if we do it and then get into the habit of sending patients to a service and then it disappears, I think that’s a bit frustrating.* ST009 (GP)

Two patients were dissatisfied with certain aspects. One felt the message was ‘the longer you are out of work, the worse it gets’ and believed they were being pushed back before being psychologically ready. The other felt the OT had an inflexible approach.

However, on balance, the majority were enthusiastic. Several wanted the OTVoc criteria widened to include unemployed people. Some felt that diagnostic labels in the eligibility criteria were unhelpful: it was often difficult to define an MSK or mental health problem and, in reality, many patients had both physical and psychological problems.

## Discussion

The study was feasible to conduct both in terms of delivering the actual intervention in primary care and in terms of conducting the research components. The data collected from both the quantitative and qualitative studies suggested that the OTVoc service was generally viewed positively and the feedback suggests individuals found it helpful in getting back to work. There was also a suggestion from staff that the clinics reduced the GP workload: however, this would need to be tested formally in a definitive trial. It was clear from both the qualitative and quantitative study results that using diagnostic labels was not helpful; it was often difficult to define the terms musculoskeletal disorder and mental health problem and, in reality, many people had both physical and psychological problems. There was support from participants to include patients with any condition and those not currently in employment. The findings suggest there is scope to do this and to widen the eligibility for OTVoc. There was also support for self-referral, particularly from the OTs.

With regard to the outcome measures used, despite the relatively small numbers, over the study period, sickness absence reduced from 50 to 17% and most of the self-reported scales indicated a slight improvement. This was notable in perceived work ability, mental wellbeing and general health. This suggests that these scales would be suitable outcome measures for a definitive trial. Moreover, the data obtained through conducting the interviews also supported these results: healthcare professionals, patients and employers were largely positive and they clearly believed that the clinics had been effective in getting patients back to work or in preparing them to return. Overall, this research lays the foundations for further trials, studies and evaluation in this area.

We specifically examined the use of the AHP H&WR in this study. Those who had experience of using it believed it was a valuable tool with the potential to provide more detailed support than the fit note. However, there was frustration regarding its legal standing in comparison to the fit note and, without appropriate changes to the current fit note, this is likely to continue. In fact, in this study, there was some support for OTs to be able to sign fit notes as has been suggested by others [21, 22]

This study comes at an important and evolving time in primary care. Our findings suggest that the intention of the UK government [10] to extend AHP powers around fit note certification is feasible and indicates that this would be well received. Other changes in GP contracting [23] will include the recruitment of OTs into primary care, and underlines that this is an area ripe for further research and evaluation.

There were limitations to the study. Recruitment was lower at one centre and there was no obvious reason for this. It might have been because of the set-up in the practice, the composition of other staff or that the numbers of OTs varied across the centres. There was also generally a low response rate to the questionnaire; this might have been because the research team were unable to contact participants by phone because of the ethical conditions set, so no rapport was established with the data collection site. However, those who completed the questionnaire seemed to have few problems, and all the scales were completed appropriately. Finally, it is worth noting that interviews required much flexibility as many had to be conducted outside normal working hours, and were often rearranged at short notice by participants. There was again difficulty in contacting participants with many not answering their phones to an unknown number. There were particular issues around recruiting employers for the interviews, although this had been anticipated. It is also likely there was sampling bias in that people who felt more negatively about OTVoc, or who were busy back at work, might have been less likely to participate in the research.

## Conclusions

OTVoc was well received and showed potential for further development. There was a suggestion that OTs were better placed and had more time than GPs to deal with health-related work issues. The AHP H&WR showed promise to be used more in GP surgeries and there were some who felt OTs should be able to complete fit notes. Although OTVoc was a small feasibility study, quantitative data indicated that sickness absence decreased from 71 to 15% and use of GP fit notes reduced from 76 to 6%. Importantly, the interview data also supported these results and found that healthcare professionals, patients and employers experienced positive reductions in sickness absence and use of GP fit Notes. These results demonstrate that there is potential to develop the vocational advisory role of OTs in primary care, and that extending AHP powers around fit note certification would be feasible and well received. There is great potential to improve the current management of vocational issues in Primary Care.

## Data Availability

The datasets used and/or analysed during the current study are available from the corresponding author on reasonable request.

## Abbreviations

AHP: Allied Health Professional
AHP H&WR: Allied Health Professions Health and Work Report
GP: General practitioner
MSK: Musculo-skeletal
OT: Occupational therapist
OTVoc: Occupational therapy-led vocational clinic

## References

[1] Waddel G, Burton AK (2006) Is Work Good for Your Health and Well-Being? DWP Independent Review. https://www.gov.uk/government/publications/is-work-good-for-your-health-and-well-being (accessed 3 Sept 2020).

[2] Black C (2008). Dame Carol Black’s review of the health of Britain’s working age population: Working for a Healthier Tomorrow.

[3] Black C, Frost D (2011). Health at work – an independent review of sickness absence. DWP November 2011.

[4] Waddell G, Burton AK, Kendall NAS (2008). Vocational rehabilitation. What works, for whom, and when?.

[5] Department for Work and Pensions 2010. Statement of Fitness for work a guide for occupational health professionals https://www.gov.uk/government/publications/fit-note-guidance-for-occupational-health-professionals (accessed 3 Sept 2020).

[6] NHS Digital (2017). Fit notes issues by GP practices, April to June 2017. Available from: https://digital.nhs.uk/catalogue/PUB30123 (accessed 14 Sept 2020).

[7] Coole C, Nouri F, Potgieter I (2015). Completion of fit notes by GPs: a mixed methods study. Perspect Public Health.

[8] Coole C, Potgieter I, Nouri F (2015). Return-to-work outcomes and usefulness of actual fit notes received by employers. Fam Pract.

[9] Allied Health Professions Federation. What is the AHP health and work Report? (2019) http://www.ahpf.org.uk/AHP_Health_and_Work_Report.htm (accessed 08 Sept 2020).

[10] Department for Work and Pensions (2017). Improving Lives: The Future of Work, Health and Disability https://assets.publishing.service.gov.uk/government/uploads/system/uploads/attachment_data/file/663399/improving-lives-the-future-of-work-health-and-disability.PDF (accessed 16 Sept 2020).

[11] Wynne-Jones G, Artus M, Bishop A, et al. Effectiveness and costs of a vocational advice service to improve work outcomes in patients with musculoskeletal pain in primary care: a cluster randomised trial (SWAP trial ISRCTN 52269669). Pain. 2018;159(1):128–8. 10.1097/j.pain.0000000000001075 28976423

[12] Ebrahim S, Malachowski C, Kamal M (2015). Measures of patients’ expectations about recovery: a systematic review. J Occup Rehabil.

[13] Ahlstrom L, Grimby-Ekman A, Hagberg M (2010). The work ability index and single-item question: associations with sick leave, symptoms, and health--a prospective study of women on long-term sick leave. Scand J Work Environ Health.

[14] Schwarzer R, & Jerusalem M (1995) Generalized self-efficacy scale. In J. Weinman, S. Wright, & M. Johnston, measures in health psychology: a user’s portfolio. Causal and control beliefs (pp. 35-37). Windsor, UK: NFER-NELSON.

[15] Tennant R, Hiller L, Fishwick R (2007). The Warwick-Edinburgh mental well-being scale (WEMWBS): development and UK validation. Health Quality Of Life Outcomes.

[16] Brooks R, on behalf of the EuroQol Group (1996). EuroQol: the current state of play. Health Policy.

[17] Herdman M, Gudex C, Lloyd A (2011). Development and preliminary testing of the new five-level version of EQ-5D (EQ-5D-5L). Qual life res.

[18] Franche RL, Corbière M, Lee H (2007). The readiness for return-to-work (RRTW) scale: development and validation of a self-report staging scale in lost-time claimants with musculoskeletal disorders. J Occup Rehabil.

[19] Lagerveld SE, Blonk RWB, Brenninkmeijer V (2010). (2010) return to work among employees with mental health problems: development and validation of a self-efficacy questionnaire. Work Stress.

[20] Ritchie J, Lewis J (2003). Qualitative research practice: a guide for social science students and researchers.

[21] Thomson L, Hampton R. Fit for work? Changing fit note practice among GPs. Brit J Gen Pract. 2012;62(595):e147–e150. 10.3399/bjgp12X625300. 10.3399/bjgp12X625300 PMC3268495 22520793

[22] BMA Report. Trust GPs to lead: learning from the responses to COVID-19 within general practice in England. British Medical Association 2020. https://www.bma.org.uk/advice-and-support/covid-19/bma-asks/trust-gps-to-lead-learning-from-the-response-to-covid-19-within-general-practice-in-england.

[23] NHS England & the British Medical Association. Update to the GP contract agreement 2020/21 – 2023/24. [online] NHS England, 6th February 2020, retrieved on 14 September 2020 https://www.england.nhs.uk/publication/investment-and-evolution-update-to-the-gp-contract-agreement-20-21-23-24/.

